# Feeling ‘too fat’ rather than being ‘too fat’ increases unhealthy eating habits among adolescents – even in boys

**DOI:** 10.3402/fnr.v60.29530

**Published:** 2016-02-16

**Authors:** Jolanda S. van Vliet, Per A. Gustafsson, Nina Nelson

**Affiliations:** 1 Medical Faculty, Department of Clinical and Experimental Medicine, Division of Paediatrics, Linköping University, Linköping, Sweden; 2 Medical Faculty, Department of Clinical and Experimental Medicine, Division of Child and Adolescent Psychiatry, Linköping University, Linköping, Sweden

**Keywords:** body image, overweight, adolescent behaviour, food habits, prevention

## Abstract

**Background:**

Adolescence is a period of gender-specific physical changes, during which eating habits develop. To better understand what factors determine unhealthy eating habits such as dieting to lose weight, skipping meals, and consumption of unhealthy foods, we studied how physical measurements and body perception relate to eating habits in boys and girls, before and during adolescence.

**Methods:**

For this cross-sectional study, we obtained data from both written questionnaires and physical measurements of height, weight, and waist circumference (WC).

**Results:**

Dieting to lose weight and skipping breakfast were more common among adolescents than among younger boys and girls (*p<*0.05). The strongest risk factor for dieting in both boys and girls was perception of overweight, which persisted after adjusting for age and for being overweight (*p<*0.01). Another independent risk factor for dieting behaviour was overweight, as defined by body mass index (BMI) among boys (*p<*0.01) and WC among girls (*p<*0.05). In both boys and girls, skipping breakfast was associated with both a more negative body perception and higher BMI (*p<*0.05). Skipping breakfast was also associated with age- and gender-specific unhealthy eating habits such as skipping other meals, lower consumption of fruits and vegetables, and higher consumption of sweets and sugary drinks (*p<*0.05).

**Conclusion:**

Body perception among adolescents is an important factor relating to unhealthy eating habits, not only in girls, but even in boys. Focus on body perception and eating breakfast daily is crucial for the development of healthy food consumption behaviours during adolescence and tracking into adulthood.

Unhealthy food consumption patterns and dieting behaviours in children and adolescents have become a major public health concern. In the developed world, skipping breakfast, low consumption of fruits and vegetables, and high consumption of sweets and soft drinks are common among adolescents (1, 2). Eating habits among children and adolescents are of special concern because behaviours adopted early in life are likely to persist into adulthood (3, 4). Moreover, overweight and obese children commonly become overweight and obese adults, with increased risk of illnesses (5).

Unhealthy eating habits are associated with both obesity and weight dissatisfaction (6, 7). The prevalence of obesity has increased in both affluent and non-affluent countries. Also in Sweden, an increase in body mass index (BMI) among age groups ranging from early childhood to late adolescence has been reported (8). An inverse association has been found between BMI and meal frequency, suggesting that obese individuals try to lose weight by skipping meals (9, 10). At the same time, the attempts by adolescents to lose weight are not only strongly defined by weight status, but also by gender, age, and perception of overweight (10). Perception of overweight is more common among girls than among boys and because girls have been shown to eat breakfast less often than boys, skipping breakfast may be part of their weight control behaviours (1, 11, 12).

To better understand what factors determine unhealthy eating habits, we studied how physical measurements and body perception relate to eating habits in boys and girls, before and during adolescence.

## Methods

### Participants

Children and adolescents aged 7–17 at selected schools in Växjö, Sweden, were invited to participate (*n*=391). In all, 67% of all students (*n*=261), including 60% of the boys (*n=*104) and 73% of the girls (*n*=157), participated during the autumn of 2010 as part of a larger research project.

### Questionnaire and anthropometry

The participants completed a questionnaire in the classroom. This questionnaire consisted of different packets of questions, in which the sections concerning body perception and eating habits were relevant for this study. These questions were posed as in the international World Health Organization survey on Health Behaviour in School-aged Children (HBSC) (13). To assess body perception, we asked the HBSC question: ‘Do you think your body is …’. Response categories on a five-point Likert scale ranged from ‘far too thin’ to ‘far too fat’. Perception of overweight was defined responding ‘too fat’ or ‘far too fat’.

Eating habits were defined as the ways in which individuals select, combine, and consume meals and foods. We defined dieting behaviour responding ‘Yes, I am on a diet’ or ‘No, but I would need to lose weight’ on the HBSC question ‘Are you currently on a diet to lose weight?’ Meal and food consumption were obtained by asking students how frequently they consumed breakfast, lunch, dinner, snacks, fruits, vegetables, sweets, and sugary drinks on weekdays, using Likert scales as in the HBSC study. Skipping meals was defined as consumption of main meals less than five times during a school week.

Physical measurements of height, weight, and waist circumference (WC) were made at the selected primary and secondary schools, by the ordinary school nurses trained by the researchers and supplied with detailed written instructions. Individual measurements were obtained using calibrated school equipment. Height was measured to the nearest 0.1 cm and weight to the nearest 0.1 kg. WC was measured to the nearest 0.1 cm midway between the 10th rib and the iliac crest. The children were measured wearing light clothing, t-shirt, and trousers, but without shoes and belts. All measurements were taken in school during school hours. No interindividual consistency analyses were performed.

### Data analysis

The physical measurements were used to calculate BMI (BMI=weight/height^2^). Weight status of the adolescents was categorized using age- and gender-specific cut-off points for BMI, the socalled IsoBMI, for overweight (IsoBMI 25) and obesity (IsoBMI 30) respectively, as defined by Cole et al. (14). To determine the age- and gender-specific cut-off points for overweight according to WC, we used the percentiles developed by McCarthy et al. (15).

We defined younger children as age 7–12.99 years and older children as ≥13 years. The age 13 cut-off enabled us to compare children (mean age 10.4±1.5 years; *n=*148) with adolescents (mean age 15.0±0.9 years; *n=*113), while also allowing for comparison between children attending primary school and secondary school.

Data were entered into the SPSS 22.0 statistics program. For gender and age differences, we used the chi-square test for non-parametric variables. To study the gender-specific correlations relating eating habits to actual body size, body perception, and age, as well as associations within the different consumption behaviours, Spearman's rho coefficients were calculated separately for boys and girls. We used univariate logistic regression modelling to study the exact odds ratios (OR) for both overweight and perception of overweight in relation to unhealthy eating habits. Multiple regression modelling was used to adjust the OR for unhealthy eating habits in relation to perception of overweight, overweight, and age ≥13. Because of attrition, the number of subjects in separate analyses varied depending on what variables were included. Level of significance was set at *p<*0.05 for all analyses.

### Ethical considerations

This study was part of a larger research project, which was preceded by written information provided at the school to all participating children and their families concerning the purpose of the research, voluntary participation, consent, and confidentiality. Written parental informed consent was obtained from all parents and from the children in seventh grade (12–13 years old) and up. The study was approved by the Regional Ethical Review Board in Linköping D-nr 07-182.

## Results

Table 1 shows the prevalence of boys and girls who demonstrated dieting behaviours and skipped main meals, in relation to age. Among boys, a higher percentage of boys ≥13 years skipped breakfast and dinner, compared with boys <13 years (*p<*0.05). Among girls, no age difference was found for the variable ‘being on a diet’, but for ‘feeling the need to lose weight’ (*p<*0.001). Feeling the need to lose weight was significantly higher among girls than among boys (*p<*0.01). Girls ≥13 years were more likely to skip breakfast and lunch than girls <13 years (*p<*0.05). No significant gender differences were found in the prevalence of skipping breakfast, lunch, or dinner.

**Table 1:** Prevalence of dieting and skipping meals on weekdays among boys and girls, classified according to age

				Boys (*n=*95)			Girls (*n=*138)		
					
Unhealthy eating habits	Boys (*n=*95)	Girls (*n=*138)	*p*	<13 years (*n=*62)	≥13 years (*n=*33)	*p*	<13 years (*n=*72)	≥13 years (*n=*66)	*p*
Dieting									
On a diet	1.2%	5.8%	0.106	2.0%	0.0%	0.440	4.6%	7.9%	0.493
Feels the need to lose weight	8.4%	20.3%	0.010	11.5%	2.9%	0.151	8.5%	33.9%	0.000
Skipping meals weekdays									
Skipping breakfast	9.5%	15.2%	0.147	4.8%	18.2%	0.034	5.6%	25.8%	0.001
Skipping lunch	5.2%	8.0%	0.621	6.5%	2.9%	0.459	2.7%	13.8%	0.016
Skipping dinner	17.7%	14.4%	0.556	9.7%	32.4%	0.005	12.3%	16.7%	0.467

The ORs for dieting and skipping breakfast when perceived or measured overweight are presented in Table 2 for boys and girls, respectively. Among girls, the risk of being on a diet increased significantly with perception of overweight and persisted after adjusting for measured overweight according to IsoBMI and WC, as well as for age ≥13. Moreover, measured overweight according to IsoBMI was an independent risk factor for girls to be on a diet (*p<*0.05), because it persisted even after adjusting for other factors. Feeling the need to lose weight increased significantly with perception of overweight among both girls and boys (*p<*0.01), also after adjusting for other factors (*p<*0.01). Measured overweight according to IsoBMI among boys (*p<*0.01) and WC among girls (*p<*0.01) were other significant independent gender-specific risk factors. The risk for skipping breakfast increased with perception of overweight among girls only (*p<*0.01) and persisted after adjusting for the other factors (*p<*0.05).

**Table 2:** Logistic univariate regression model predicting odds ratio (OR) and confidence interval (CI) for determinants of unhealthy eating habits

	OR (CI)	OR (CI) adjusted for overweight perception	OR (CI) adjusted for overweight IsoBMI^a^	OR (CI) adjusted for overweight WC^b^	OR (CI) adjusted for age ≥ 13 years
**Boys (*n*=106)**					
On a diet					
Perception overweight	0.0^c^				
Overweight IsoBMI^a^	0.0^c^				
Overweight WC^b^	0.0^c^				
Need to lose weight					
Perception overweight	152.3** (14.9–1553.4)		63.4** (4.1–988.4)	98.3** (9.0–1072.4)	134.1** (12.9–1395.6)
Overweight IsoBMI^a^	96.8** (10.2–921.6)	39.4** (2.5–626.1)		105.7** (5.4–2067.4)	92.1** (9.4–904.1)
Overweight WC^b^	12.7** (2.4–68.5)	5.5 (0.6–51.4)	0.9 (0.06–13.1)		10.9** (2.0–59.5)
Skipping breakfast					
Perception overweight	3.8 (0.8–17.4)		2.7 (0.4–18.8)	3.0 (0.6–16.3)	8.8* (1.3–58.2)
Overweight IsoBMI^a^	3.0 (0.7–13.2)	1.7 (0.2–11.5)		2.3 (0.3–16.0)	5.4 (1.0–30.0)
Overweight WC^b^	2.2 (0.6–8.7)	1.5 (0.3–7.1)	1.4 (0.2–8.6)		4.4 (0.9–21.1)
**Girls (*n*=147)**					
On a diet					
Perception overweight	51.7** (5.4–491.9)		21.8* (1.9–244.6)	35.5** (3.5–361.4)	80.3** (6.2–1035.2)
Overweight IsoBMI^a^	39.1** (4.2–366.1)	16.6* (1.5–187.9)		0.0^c^	46.0** (4.6–461.0)
Overweight WC^b^	12.8* (1.4–114.5)	8.0 (0.7–86.4)	0.0^c^		14.2* (1.5–130.6)
Need to lose weight					
Perception overweight	68.0** (19.3–239.5)		64.2** (16.9–243.3)	56.7** (14.8–217.4)	49.4** (13.5–180.8)
Overweight IsoBMI^a^	3.6* (1.3–10.2)	0.9 (0.2–3.9)		1.4 (0.4–4.6)	6.2** (1.8–21.2)
Overweight WC	5.2** (2.1–13.0)	4.9* (1.3–18.1)	4.5** (1.6–13.1)		7.1** (2.6–19.8)
Skipping breakfast					
Perception overweight	4.5** (1.8–11.2)		4.6** (1.6–12.8)	3.8* (1.4–10.6)	3.1* (1.1–8.7)
Overweight IsoBMI^a^	2.4 (0.8–6.9)	1.3 (0.4–4.2)		1.5 (0.4–5.7)	3.2 (1.0–10.7)
Overweight WC^b^	2.5 (1.0–6.6)	1.9 (0.7–5.1)	2.1 (0.6–6.8)		3.0* (1.1–8.2)

a Age and gender-specific body mass index (BMI) for children up to 18 years;

b waist circumference (WC);

c OR was zero because of one empty cell.

* *p<*0.05,

** *p<*0.01.

Table 3 shows how eating habits among boys and girls correlate with body perception, BMI, WC, and age. In boys, body dissatisfaction and higher BMI were inversely correlated with frequency of breakfast on weekdays (*p<*0.05). BMI and WC were negatively correlated with consumption of vegetables (*p<*0.05), but positively correlated with consumption of sugary drinks (*p<*0.01). In boys, frequency of dinner and consumption of vegetables were negatively correlated with age, whereas consumption of sweets and sugary drinks showed a positive relationship with age (*p<*0.05). Among girls, frequency of breakfast and lunch was negatively related to body dissatisfaction, higher BMI, WC, and age (*p<*0.05), whereas body dissatisfaction was positively related to age (*p<*0.001) (Table 3). Body dissatisfaction, WC, and age were also negatively correlated with fruit consumption (*p<*0.05), whereas consumption of snacks showed a positive relationship with age among girls (*p<*0.05).

**Table 3:** Spearman's coefficients for correlations between food consumption and body dissatisfaction, BMI, WC, and age in boys (*n=*106) and girls (*n=*147)

	Boys (*n=*106)				Girls (*n=*147)			
		
Consumption of:	Body perception	BMI^a^	WC^b^	Age	Body perception	BMI^a^	WC^b^	Age
Breakfast	−0.208*	−0.227*	−0.187	−0.141	−0.207*	−0.275**	−0.274**	−0.198*
Lunch	−0.166	−0.110	−0.081	−0.116	−0.127	−0.182*	−0.173*	−0.109
Dinner	0.026	−0.181	−0.187	−0.247*	−0.057	0.031	−0.010	0.017
Snacking	−0.094	−0.152	−0.169	−0.134	−0.113	−0.084	−0.064	−0.192*
Fruit	−0.180	−0.186	−0.156	−0.131	−0.206*	−0.135	−0.170*	−0.364**
Vegetables	−0.011	−0.277**	−0.234*	−0.261*	−0.156	−0.089	−0.075	−0.099
Sweets/candy	−0.017	−0.069	−0.092	0.255*	0.049	0.014	0.020	0.099
Sweet drinks	−0.099	0.261**	0.277**	0.436**	0.019	0.162	0.146	0.090

a Body mass index;

b waist circumference.

* *p<*0.05;

** *p<*0.01.

Table 4 shows consumption patterns among boys and girls, respectively, by marking the significant correlations (with stars), and indicated that food consumption patterns for boys and girls differed, although breakfast was central for both sexes. Among boys, consumption of breakfast was positively correlated with consumption of lunch and negatively correlated with consumption of sweets on weekdays (*p<*0.05).

**Table 4:** Spearman's rho coefficients for correlations within food consumption behaviours, as well as food consumption patterns marked as significant cells in boys and girls, respectively

	Boys (*n=*100)						
	
	Breakfast	Lunch	Dinner	Snacking	Fruit	Vegetables	Candy
Lunch	0.221*	1.000					
Dinner	0.113	0.252*	1.000				
Snacking	0.122	0.203*	0.118	1.000			
Fruit	0.009	0.139	0.092	0.321**	1.000		
Vegetables	0.197	0.131	0.030	0.131	0.436**	1.000	
Candy	−0.208*	−0.003	−0.001	−0.110	−0.159	−0.228*	1.000
Sweet drinks	−0.188	0.171	0.028	−0.126	−0.180	−0.225*	0.440**
							
	Girls (*n=*146)						
	
	Breakfast	Lunch	Dinner	Snacking	Fruit	Vegetables	Candy

Lunch	0.575**	1.000					
Dinner	0.235**	0.201*	1.000				
Snacking	0.165	0.182*	0.368**	1.000			
Fruit	0.191*	0.117	0.090	0.145	1.000		
Vegetables	0.174*	0.119	0.261**	0.266**	0.455**	1.000	
Candy	−0.176*	−0.092	−0.062	−0.179*	−0.032	−0.135	1.000
Sweet drinks	−0.173*	0.143	0.042	−0.107	0.007	−0.033	0.292**

* *p<*0.05,

** *p<*0.01.

In boys, lunch consumption was, in turn, positively related to consumption of dinner and to snacking behaviour (*p<*0.05). Snacking was positively correlated with fruit consumption (*p<*0.01), which in turn was positively correlated with consumption of vegetables (*p<*0.01). Consumption of vegetables was negatively related to consumption of sweets and sugary drinks (*p<*0.05), whereas consumption of sweets showed a strong positive correlation with consumption of sugary drinks in boys (*p<*0.01).

Among girls, consumption of breakfast was positively associated with consumption of lunch and dinner (*p<*0.01). Moreover, breakfast showed a direct positive correlation with consumption of fruits and vegetables, and a negative correlation with consumption of sweets and sugary drinks (*p<*0.05). Consumption of lunch was, in turn, positively related to dinner and snacking behaviour (*p<*0.05). In girls, dinner, snacking behaviour, and consumption of vegetables were positively correlated with each other (*p<*0.05), whereas snacking behaviour and consumption of sweets showed a negative association (*p<*0.05). We found that consumption of fruit was positively related to consumption of vegetables, whereas consumption of candy was positively related to sugary drinks in girls (*p<*0.01).

## Discussion

The strongest risk factor for dieting behaviours in both girls and boys was perception of overweight, which persisted after adjusting for actual overweight and age. Moreover, body dissatisfaction was associated with less frequent consumption of breakfast in both sexes. Consistent with other studies, eating habits seemed to have stronger associations with weight satisfaction than with actual body weight, especially in girls (6, 16, 17).

Girls who wished to be thinner skipped breakfast and other meals more often than girls who were satisfied with their bodies. Attitudes among girls regarding an ideal thinner body make them more likely to engage in various forms of weight reduction behaviours, including skipping breakfast (1, 9, 11, 18). Furthermore, in this study, we found that girls were more likely to feel the need to lose weight than boys, while an earlier study in the same population showed that girls were more likely to have an unrealistic perception of themselves as overweight than boys (19).

In addition to perception of overweight, another independent risk factor in girls for dieting was overweight according to WC. Meanwhile, both higher WC and BMI were shown to be associated with less frequent consumption of breakfast in girls. WC was previously found to be more strongly associated with body perception than IsoBMI among girls, possibly because of its correlation with abdominal fat at different ages (20). Girls may see the increase in weight and body fat mass caused by physical development as an obstacle to reaching their ideal of a thin female body (21, 22). Physical development in pubertal maturation might therefore explain why attempts to lose weight were more prevalent in the older age group of girls (10), a finding also confirmed in this study. Meanwhile, higher BMI and WC, but not higher age, were found to be related to less frequent consumption of lunch. In Sweden, school lunches are free on weekdays, which might imply that our findings are either an effect of peer influence or a dieting behaviour based on a more realistic perception of body size, independent of age. An interesting observation in this context and discussion on dieting behaviour in girls was the finding of less fruit consumption among those with higher body dissatisfaction and WC. Based on our results, we may infer that fruit consumption along with snacking frequency might be a part of female peer-influenced adolescent behaviour.

In contrast to girls, among boys, overweight according to IsoBMI, but not to WC, was found to be another independent risk factor for feeling the need to lose weight. This could imply that dieting or weight reduction behaviour among boys may be the result of a more realistic body perception based on an actual higher weight and BMI. An additional finding in boys was that less frequent breakfast consumption was associated with higher BMI, but not with WC. Other cross-sectional studies also found an inverse relationship between breakfast consumption and BMI, including the analysis of data from 41 countries participating in the HBSC study (23). At the same time, in boys, higher BMI and WC were found to be associated with lower consumption of vegetables and higher consumption of sugary drinks, lending credence to the idea that eating habits are the cause, rather than the effect, of body size. Adolescence, with its peer influences, is another factor that needs to be taken into account, because not only lower vegetable consumption and higher consumption of sugary drinks were related to higher age, but also skipping dinner.

While other studies have reported that girls skipped breakfast more often than boys (1), we only found age differences within each gender in breakfast behaviours, suggesting that skipping breakfast is merely a part of adolescent behaviour. Vereecken et al. also found lower daily breakfast consumption in older children than in 11-year-olds, which may be rooted in important changes that accompany adolescence, including greater autonomy and independence in food choices, decreased frequency of family meals, and increased peer influence, as well as an increase in dieting behaviour, especially among girls (1). Skipping breakfast was related to other unhealthy eating habits such as skipping other meals, lower consumption of fruits and vegetables, and higher consumption of sweets and sugary drinks in both boys and girls, consistent with the consumption patterns found in most other HBSC countries (1). Because our study also found that breakfast consumption is inversely related to both BMI and consumption of sweets, regular breakfast consumption should be integrated into prevention strategies for overweight and obesity (9).

Even though breakfast consumption was found to be associated with other eating habits in both boys and girls, the relationship between breakfast and other food consumption differed between boys and girls. In boys, a correlation chain was found, where consumption of breakfast was related to lunch, which in turn was related to snacking, and ultimately to fruit and vegetable consumption. The latter, in turn, was inversely related to consumption of sweets and sugary drinks. In contrast, among girls, breakfast consumption was directly and positively associated with consumption of lunch, dinner, fruit, and vegetables and directly but negatively associated with consumption of sweets and sugary drinks. A correlation chain similar to that seen among boys was also found among girls, differing only in the consumption pattern for vegetables in girls and for fruit in boys. One hypothesis to explain these gender differences in food consumption patterns might be that the correlation chain found in both boys and girls could be age- and peer-related, whereas the pattern of direct correlations seen in girls could be part of dieting behaviour. Further larger and longitudinal studies are required to confirm this hypothesis.

This study analysed only cross-sectional data from boys and girls, suggesting important results on associations between variables and comparisons between groups, although by nature of the study design, the results are unable to clarify any possible cause-effect chains. In addition, the study population was relatively small, which could make our results difficult to generalize. However, use of the questions as formulated in the HBSC study, which is conducted every 4 years in most European countries, allowed us to make international comparisons, as well as certain generalizations from our results. Measurement of weight, height, and WC, only feasible in small study populations, instead of self-reports in the HBSC study was a strength of our study. Another was the inclusion of boys as little is known about their body perception and even less concerning any associations with eating habits. However, body perception in both boys and girls was measured with a single-item categorical response, which may be imprecise. Despite this potential limitation, a single-item measure may also be favourable because it is able to roughly indicate unhealthy perceptions and eating habits. Moreover, a single-item approach to evaluation of body perception can easily be assessed both on the individual level and in large-scale screening programmes, and thus be of great value in the prevention of unhealthy eating habits and resultant ill health and disease.

We conclude that body perception and breakfast consumption are important in the development of healthy eating habits during adolescence, tracking into adulthood. To benefit the future health and health behaviours of school children, we wish to emphasize first of all the concept of body perception and suggest educational interventions to improve understanding of the physical changes that normally accompany growth and pubertal development, and discuss with children and adolescents how they perceive these bodily changes. We also suggest focus on breakfast consumption to assess and discuss the risk for other unhealthy eating habits as well as to help prevent overweight and obesity. Action for better eating habits should be taken both at the individual level and at the societal level by creating conditions, attitudes, interventions, and programmes for healthy meals and eating habits at the different arenas where young people are found, such as at home, in school, at leisure time, and also in their living environment and neighbourhood.
